# Classification of vascular anomalies

**DOI:** 10.1308/rcsann.2025.0106

**Published:** 2026-01-12

**Authors:** V Sahni

**Affiliations:** All India Institute of Medical Sciences New Delhi, India

I write further to a recent paper in The *Annals* entitled ‘Rare case of combined vascular malformation of the face: a successful surgical management’.^1^

The authors report a ‘revised’ International Society for the Study of Vascular Anomalies (ISSVA) classification; however, the cited literature and indeed the description are based on the 2014 version.^1^ They report that the malformations are divided into simple and combined,^1^ whereas in the 2014 version two further divisions of malformations were described, namely that of major named vessels and those associated with other anomalies.^2^

This classification has undergone subsequent revisions (even prior to the reporting of this paper). The 2018 revision divided vascular anomalies into vascular tumours and vascular malformations.^3^ The tumours were further divided into benign, borderline/locally aggressive or malignant.^3^ The malformations were divided into simple (capillary, lymphatic, venous, arteriovenous and arteriovenous fistula), combined (CVM, CLM, CAVM, LVM, CLVM, CLAVM, CVAVM and CLVAVM; C: Capillary, V: Venous, AV: Arteriovenous, M: Malformation), anomalies of major named vessels/truncal/channel type (affecting: arteries, veins or lymphatics; anomalies of: length, number, course, origin, persistence, communication, valves or diameter) and associated with other anomalies.^3^

The 2025 revision (Figure 1) adds the category of PUVA (Potentially Unique Vascular Anomaly) to tumours and malformations and retains the tumour subdivisions.^4^ Vascular malformations are further divided into fast-flow (isolated, multifocal or syndromic), slow-flow (capillary, lymphatic, venous or combined) and developmental anomalies pertaining to named vessels.^4^

**Figure 1:**
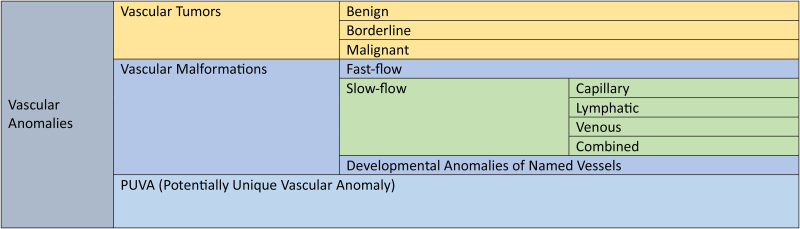
An overview of the 2025 ISSVA Classification for vascular anomalies

## Competing interests

The author/s declare no competing interests.

## Funding

The author/s received no financial support for the research, authorship and/or publication of this article.

## Author contributions

**V Sahni**: Conceptualisation, Data curation, Formal analysis, Investigation, Methodology, Project administration, Resources, Software, Supervision, Validation, Visualisation, Writing – original draft, Writing – review & editing.

## Artificial Intelligence

The author/s declare that no AI was used to conduct the study or prepare the manuscript.
